# Depression among Tibetan residents in the Southeastern region of Qinghai-Tibet plateau: a cross-sectional study

**DOI:** 10.1038/s41598-024-84357-5

**Published:** 2025-01-02

**Authors:** Yuji Chen, Ga Long, Qing Huang, Ping Zhang, Nie Xu

**Affiliations:** 1 Department of Oncology, Chengdu First People’s Hospital, Chengdu, China; 2 Internal Medicine, Dege County People’s Hospital, Ganzi Tibetan Autonomous Prefecture, Sichuan, China; 3 Department of Radiotherapy, Sichuan Cancer Center, Sichuan Cancer Hospital & Institute, Chengdu, China

**Keywords:** Tibetan, Depression, High altitude, Qinghai-Tibet plateau, Physiology, Psychology, Diseases, Health care, Risk factors

## Abstract

Depression has emerged as a significant public health concern, with its prevalence fluctuating based on varying environmental and demographic factors. This study categorized participants based on altitude. A convenient sampling approach was used, and the hamilton depression rating scale-24 was used to assess depressed symptoms while gathering demographic information. A total of 600 Tibetan residents from the Dege area of Garze Prefecture, Sichuan, China, participated in the survey. The mean age is 56.81 years, males comprising 52.8% and females 47.2% of the sample. Of the participants, 41.2% resided permanently at elevations exceeding 3500 m. The results found that the weighted prevalence of depression in the area was 24.62%. Regardless of gender, the age group with the highest prevalence was 55–64 years old. Depression increased with age as well as gradually decreased after the age of 60. Logistic regression analysis showed that middle-aged (OR 2.86, 95% CI 1.69–4.82, *P* < 0.01) and elderly people (OR 2.27, 95% CI 1.30–3.98,* P* < 0.01), living in ultra-high altitude areas (OR 3.48, 95% CI 1.35–2.91,* P* < 0.01) and low BMI (OR   4.31, 95% CI 1.33–13.93) are high-risk factors for depression. This study enhances the understanding of the characteristics of depression in high-altitude regions of China, contributing to a more comprehensive view of the psychological well-being of residents in these areas. The findings underscore the need for targeted prevention and treatment strategies tailored to the specific needs of these populations.

## Introduction

Depression is a major global health challenge that has garnered significant attention. According to the world health organization (WHO), more than 350 million people around the world suffer from this disorder, ranking it as the fourth primary cause of disease globally, with prevalence rates rang ing from 3 to 5%^1^. Numerous geographic locations and lifestyle choices have been shown to affect the prevalence of depression. Altitude is a notable environmental determinant^2^. The prevalence and probability of depression or depressed symptoms are higher among people who live at higher elevations than among people who live in low-lying areas^3^. Due to China’s vast geographical size and significant variation in elevation, a large portion of its ethnic minority populations reside in ultra-high-altitude regions above 3500 m. Despite the high prevalence of depression in China, previous research has primarily focused on low-altitude areas, resulting in limited data on the prevalence of depression among ethnic minority groups in high-altitude regions.

Dege County, located in Garze Prefecture, Sichuan Province, lies in the southeastern part of the Qinghai-Tibet Plateau. The region’s elevation ranges from 2980 to 6168 m, with an average of 4235 m. The local economy is mostly based on livestock husbandry, and 96% of the population identifies as Tibetan^4^. The area faces harsh natural conditions, further exacerbated by limited resources, uneven population distribution, low levels of educational attainment, restricted economic opportunities, and inadequate healthcare. As a result, these problems cause significant variations in mental health status when compared to other parts of China.

To gain a deeper understanding of depression in high-altitude regions of China, this cross-sectional study used a questionnaire survey to assess the depression state of people living on Sichuan’s Garze Plateau. Analyzing the associated risk factors will enhance our understanding of the underlying causes of depression in these areas, allowing for the creation of more effective prevention and treatment strategies.

## Methods

### Study design and setting

This study involved 600 Tibetan residents in the Dege area of Garze Prefecture, located in western Sichuan, China, and was conducted from January 2020 to June 2022. Participants were required to be at least 15 years old and have resided in Ganzi Prefecture for an extended period. Additionally, all participants identified as Tibetan. Individuals with a documented history of mental illness were excluded from the study.All participants included in the study reside at an altitude exceeding 3000 m. They were categorized into two groups based on their residential altitude: high-altitude areas (< 3500 m) and ultra-high-altitude areas (> = 3500 m). A convenience sampling approach was employed to recruit the participants. Due to the participants’ low educational levels, verbal questioning was employed. The survey was administered by eight doctors, one of whom was a psychiatrist, all of whom underwent standardized and rigorous training on the survey content. Participants who were not proficient in Chinese completed the questionnaires with the assistance of a research assistant fluent in Tibetan. All participants received free health counseling as compensation.

Before participating, we assured the participants of the privacy safeguards in place and stressed that the data would be used purely for academic reasons, with no disclosure to third parties. Informed consent was obtained from all the participants and/or their legal guardians. This study was approved by the Institutional Review Board of Dege County Peopl’s Hospital,

and adhered to the principles of the Helsinki Declaration(2013). We confrmed that all research was performed in accordance with relevant guidelines and regulations.

### Survey instruments

This research used the hamilton depression rating scale-24 (HAMD-24) to assess depressive symptoms. The hamilton depression rating scale, regarded as one of the most widely used depression evaluation tools in the world, not only measures the severity of depressed symptoms but also tracks their improvement over time. The 24-item version is an updated iteration of the original scale.In this investigation, a validated Chinese adaption of the 24-item hamilton depression rating scale (HAMD-24) was used for assessment^5^ This version has been confirmed in the Chinese demographic, with a sensitivity of 0.87 and a specificity of 0.92^6^.

The scale consists of 24 components, which are grouped in seven dimensions^7^. (1) Anxiety/Somatization, which includes five items such as psychic anxiety, somatic anxiety, gastrointestinal issues, hypochondriasis, and insight. (2) Weight, focusing exclusively on weight loss. (3) Cognitive Impairment, which includes six items such as feelings of guilt, suicidal thoughts, agitation, depersonalization, derealization, paranoid symptoms, and obsessive–compulsive behaviors. (4) Diurnal Variation, which specifically examines alterations in mood or behavior throughout the day and night. (5) Retardation, characterized by slowed thinking and speech, difficulties with concentration, and lack of initiative. (6) Sleep Disturbance, which includes three aspects such as difficulty falling asleep, unrefreshing sleep, and early morning awakenings; depression. (7) Helplessness, which includes three questions related to sentiments of low capacity, hopelessness, and inferiority. Each item is scored on a scale of 0 to 4. The HAMD-24 score aids in determining the severity of depressive symptoms: scores below 8 indicate no depression, scores between 8 and 19 indicate mild depression, scores between 20 and 34 indicate moderate depression, and scores of 35 or higher indicate severe depression^8^. A total score of 8 or higher indicates depression, with higher scores suggesting more severe depressed symptoms^9^.

### The definition and classification

(1) We defined a range of actual age at the time of investigation^10^: Youth (15–44 years), Middle-Aged (45–64 years), and Elderly (65 + years).(2) Permanent residence was categorized into urban (urbanised areas or neighborhood committee) and rural (villages or countryside) areas. (3) According to the internationally accepted altitude classification standard, high altitude is defined as geographical altitude ≥ 1500 m, and >  = 3500 m is ultra-high altitude^2^. According to the place of residence of the respondents, we divide them into those with an altitude >  = 3500 m and those with an altitude < 3500 m. All respondents lived at altitudes exceeding 3000 m. (4) Body mass index (BMI) was calculated using the formula: BMI = body weight (kg)/[height (m)]^2^.

### Statistical analysis

First, we described the general characteristics of the subjects in this study, which are presented in Table 1, including sex, the permanent residence, the altitude of the permanent residence, education, famer, smoking, alcohol consumption, and body mass index (BMI, calculated as body weight in kilograms divided by height^2^). Second, adjusted odds ratios (OR) and 95% confidence intervals (CI) were estimated by multivariable logistic modelto evaluate the differences in depression prevalence among the groups within one characteristic, with adjustment of the other characteristics in (Table 2). Besides, in order to further evaluate the relationship between age and depression prevalence, as well as BMI and depression prevalence, restricted cubic spline analyses were performed, which was added in the logistic models on the basis of age and BMI, respectively. Third, we calculated the total prevalence of depression weighted by the population quantity stratified by sex and age for Dege Countys (Table 3), using data from the 7th National Population Census. The differences in the prevalence of depression and depression scores between male and female in each age group were tested by χ^2^-test and Wilcoxon-Mann–Whitney test, respectively.All statistical analyses were performed using STATA 17.0.

**Table 1:** General characteristics of the study subjects.

Characteristics	N	%
Sex		
Male	317	52.8
Female	283	47.2
Altitude		
< 3500 m	353	58.8
> = 3500 m	247	41.2
Residence		
Rural	521	86.8
Urban	79	13.2
Educational level		
College	41	6.8
Middle school	22	3.7
Primary school	67	11.2
Illiterate	470	78.3
Famer (Y/N)		
Yes	484	79.6
No	124	20.4
Smoking (Y/N)		
No	562	93.7
Yes	38	6.3
Drinking (Y/N)		
No	542	90.3
Yes	58	9.7
Body mass index		
< 18.5	14	2.3
18.5–24.0	238	39.1
24.0–28.0	219	36.0
> = 28.0	137	22.5
Depression scores		
8–19	169	28.2
20–34	13	2.1
≥ 35	0	0

**Table 2:** Prevalence of depression and related demographic factors.

Characteristic	Depression (n = 182)								
	Number	Effective percentage	χ^2^	OR	95% CI		Adjusted OR^&^	95% CI	
Sex			1.621						
Male	89	28.1		1.00	Ref		1.00	Ref	
Female	93	32.9		1.25	0.88–1.78		1.30	0.88–1.90	
Age group			17.66**						
Youth (15–44)	30	17.9		1.00	Ref		1.00	Ref	
Middle-aged (45–64)	94	36.4		2.64	1.65–4.22		2.86	1.69–4.82	
Elderly (> = 65)	58	33.3		2.30	1.39–3.81		2.27	1.30–3.98	
Altitude			15.871**						
< 3500 m	85	24.1		1.00	Ref		1.00	Ref	
> = 3500 m	97	39.3		2.04	1.43–2.90		3.48	1.35–2.91	
Residence			0.606						
Rural	161	30.9		1.24	0.73–2.10		0.70	0.35–1.42	
Urban	21	26.6		1.00	Ref		1.00	Ref	
Education			9.374*						
College	12	29.3		0.84	0.42–1.69		1.73	0.62–4.83	
Middle school	3	13.6		0.32	0.09–1.10		0.50	0.13–1.91	
Primary school	12	17.9		0.44	0.23–0.85		0.52	0.26–1.05	
Illiterate	155	33.0		1.00	Ref		1.00	Ref	
Famer			5.248*						
Yes	157	32.4		1.75	1.08–2.83		1.77	0.85–3.67	
No	25	21.6		1.00	Ref		1.00	Ref	
Smoking			4.061*						
No	176	31.3		1.00	Ref		1.00	Ref	
Yes	6	15.8		0.41	0.17–1.00		0.57	0.21–1.55	
Drinking			0.015						
No	164	30.3		1.00	Ref		1.00	Ref	
Yes	18	31.0		1.04	0.58–1.86		1.69	0.84–3.39	
Body mass index			3.496						
< 18.5	7	50.0		2.61	0.88–7.72		4.31	1.33–13.93	
18.5–24.0	66	27.7		1.00	Ref		1.00	Ref	
24.0–28.0	69	31.5		1.20	0.80–1.79		1.04	0.68–1.60	
> = 28.0	40	31.0		1.17	0.73–1.87		1.08	0.65–1.78	

*P < 0.05. **P < 0.01. & adjust other characteristics.

**Table 3:** Depression scores and prevalence rates among participants of different genders and ages.

Age group	N	Total				Male				Female			Z	P	χ^2^	P
		Mean	Standard	%	N	Mean	Standard	%	N	Mean	Standard	%				
15–24	23	3.48	5.80	23.44^#^	6	3.33	4.50	33.33	17	3.53	6.32	11.76	0.189	0.850	1.436	0.231
25–34	56	3.55	6.22	16.84^#^	24	2.33	3.58	12.50	32	4.47	7.55	21.88	−0.180	0.857	0.822	0.365
35–44	89	3.10	5.13	18.56^#^	51	2.76	5.00	13.73	38	3.55	5.34	23.68	−0.528	0.598	1.465	0.226
45–54	131	5.26	5.95	26.10*	70	4.31	4.89	20.00	61	6.34	6.84	32.79	−1.463	0.143	2.773	0.096
55–64	127	6.86	5.66	47.76^#^	69	6.00	5.16	42.03	58	7.88	6.09	53.45	−1.669	0.095	1.649	0.199
65–74	123	5.29	4.83	31.08^#^	68	5.69	5.32	35.29	55	4.80	4.15	27.27	0.512	0.608	0.904	0.342
> = 75	51	5.33	4.93	38.47^#^	29	5.03	4.54	34.48	22	5.73	5.48	40.91	−0.125	0.900	0.221	0.638
Total	600	5.06	5.61	24.62^#^	317	4.62	5.07	23.12^#^	283	5.55	6.13	26.24^#^	−1.350	0.177	1.621	0.203

^#^Weighted prevalence of depression by population and age group in the region.

## Results

### Baseline characteristics

A total of 640 Tibetan residents were surveyed, with 40 cases eliminated due to missing information and noncompliance with scale standards, obtaining a final sample size of 600 individuals. Table 1 presents the key demographic characteristics of the participants. The male-to-female ratio is 1.12:1 (males: 317, females: 283), with men comprising 52.8% and women 47.2% of the sample. The average age is 56.81 years (SD = 15.4, range = 15–94 years). A significant proportion of respondents (78.3%) had completed primary school or less. Of the participants, 41.2% (247 people) lived permanently at elevations above 3500 m, whereas 58.8% (353 people) lived at altitudes between 3000 and 3500 m. Rural residents constituted 86.8% of the sample, with farmers accounting for 79.6% of participants. Notably, 93.7% of respondents reported that they did not smoke, while 90.3% did not consume alcohol. Furthermore, 97.7% of the respondents had a BMI (body mass index) greater than 18.5.

In this study, individuals with depression (score ≥ 8) constituted 30.3% of the total sample. Participants with mild depression (scores between 8 and 19) represented 28.2% of the sample, whereas those with moderate depression (scores 20–34) comprised 2.1%. Additionally, those with severe depression (scores ≥ 35) accounted for 0% of the participant pool.

### The association between incident depression and various potential risk factors

**Fig. 1:**
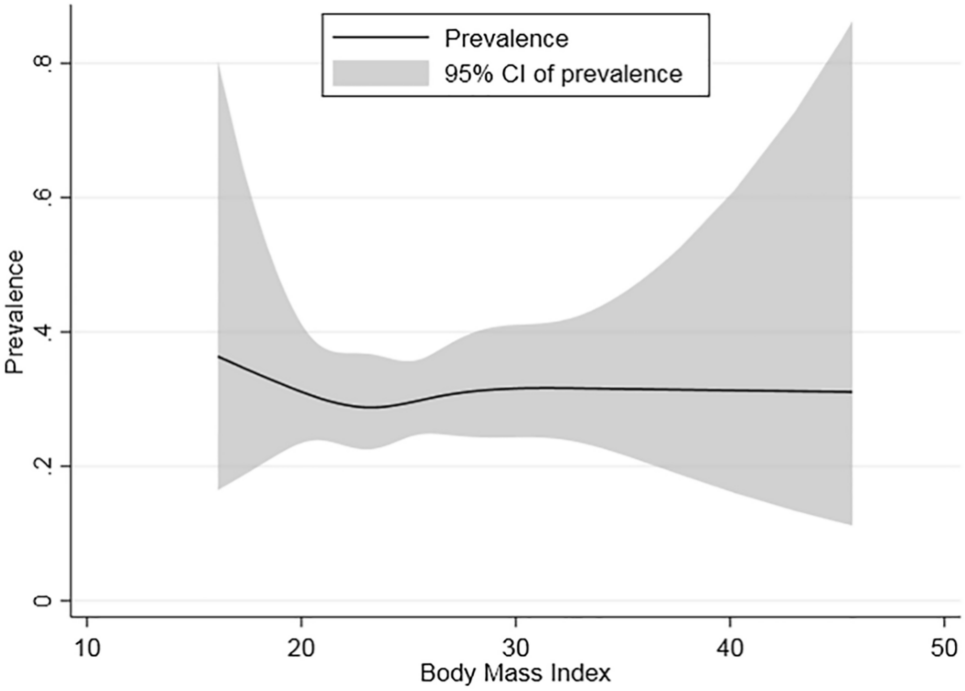
Non-linear relationship of BMI and depression.

In this research, a depression score of ≥ 8 was used to identify cases of depression (N = 182). The findings revealed a significant correlation between the prevalence of depression and factors such as age, altitude, and BMI. To further investigate the risk factors for depression associated with multiple demographic characteristics, a binary logistic regression analysis was performed on the entire sample. Research indicates that age (*P* < 0.01), altitude (*P* < 0.01), education level (*P* < 0.05), the status of being a farmer (*P* < 0.05) and smoking (*P* < 0.05) all have statistically significant impacts on depression. However, after adjusting for other variables, it was determined that age, altitude, and BMI are the primary risk factors for depression (Table 2). Notably, individuals aged 45 years and above were more prone to depression than those younger than 45, with middle-aged participants (OR 2.86, 95% CI 1.69–4.82, *P* < 0.01) and the elderly (OR 2.27, 95% CI 1.30–3.98, *P* < 0.01). Residents living at altitudes of 3500 m or higher were more likely to suffer from depression (OR 3.48, 95% CI 1.35–2.91, *P* < 0.01). Furthermore, individuals with a low body mass index (BMI < 18.5) were at an increased risk of experiencing depression in comparison to individuals who had a higher BMI (OR 4.31, 95% CI 1.33–13.93). The relationship between BMI and depression prevalence was found to be nonlinear, with an L-shaped curve (Fig. 1).

### Depression scores and prevalence rates among participants of different genders and ages

**Fig. 2:**
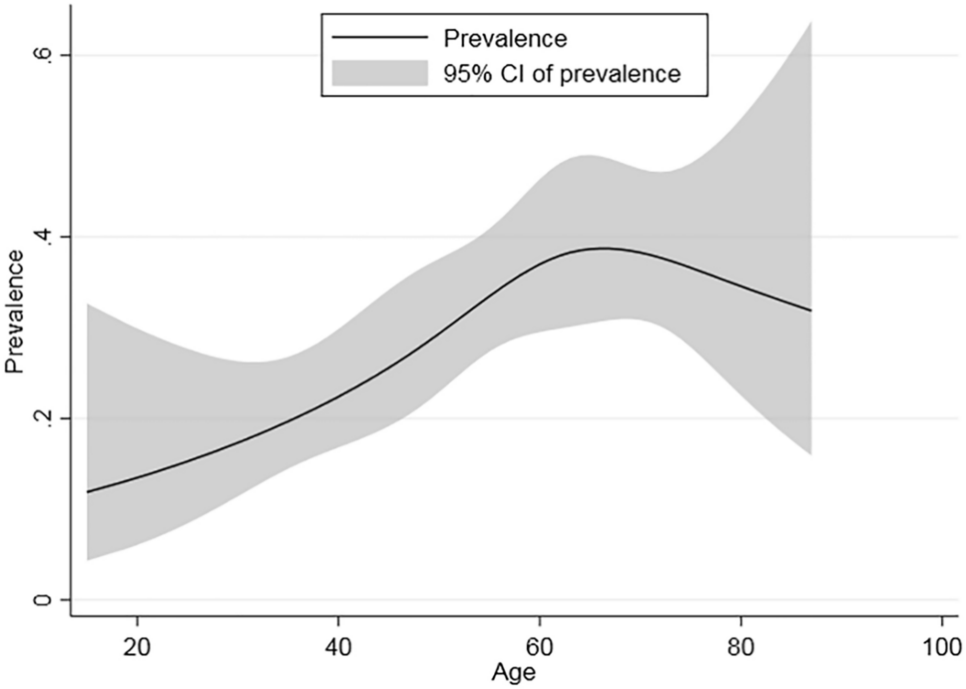
Non-linear relationship of age and depression.

To analyze gender-based differences in depression scores and prevalence rates among participants of different ages, we used t-tests and chi-square tests on male and female participants. Both males and females had the highest prevalence rates in the 55–64 age group (42.03 and 53.45%, respectively) (Table 3). Females showed a higher depression score (6.13 vs. 5.07 for males), although this difference was not statistically significant (*P* > 0.05). Our findings reveal that the incidence of depression rises with age, peaks after 60, and then declines, forming a general inverted U-shaped trajectory (Fig. 2).

## Discussion

With the advancement of socio-economic situations, there has been growing concern over emopression in low-altitude areas.However, there remains a significant lack of research on depression in high-altitude regions.

The comparatively mild environment at elevations above 1500 m makes it appropriate for human settlement and activities. The social environment is comparatively mature, and the infrastructure is well developed. However, as elevation exceeds 3500 m, the climate becomes colder and drier, with increased hypoxia and stronger ultraviolet (UV) radiation. The weather also becomes more erratic, and there is a notable difference in temperature between day and night. At this elevation, the social environment becomes increasingly challenging. The construction and maintenance of infrastructure become more complex, living conditions deteriorate, and access to medical and educational resources is limited. High-altitude areas tend to have lower population densities and higher living costs.

The findings of this study show that the rate of depression in Dege, located in Ganzi Prefecture of Sichuan Province, China, is significantly higher (24.62%) than the national rate of depression in China, which is 6.9%^11^. This increased risk of depression could be attributed to a variety of factors, including age,tional wellness. According to the 2023 National Depression Blue Paper, China has 95 million people diagnosed with depression, with an enormous increase in cases during the COVID-19 epidemic^12^. The afflicted group is becoming increasingly young, with 50% of patients being students and 30.28% being under the age of 18, totaling around 28.5 million people^13^. While most studies focus on the prevalence of de altitude, and BMI. Jiazhou Wang et al. investigated depression prevalence in the Tibetan district of Yushu, Qinghai Province. Their attention was mostly focused on the eastern half of the Qinghai-Tibetan plateau. They discovered a comparatively high depression prevalence of 28.6% in that region and hypothesized a strong positive relationship between altitude and rates of depression, which is consistent with our results. In addition, our findings indicate that people who have lived at ultra-high altitudes for an extended period of time are more susceptible to depression as the altitude increases. On the other hand, they observed that factors such as alcohol consumption, higher socioeconomic status and completing secondary education were linked to an increased risk of depression, differing from our findings.

Depression is characterized by persistent feelings of sadness and hopelessness, which might manifest as sluggish responses, reduced verbal communication, a slower rate of speech, and limited physical activity. In more severe cases, individuals may lose the ability to function entirely^14^. The causes of depression are multifactorial, including genetics, psychological trauma, lifestyle choices, environmental variables, and chronic health difficulties.

In this study, all participants lived at altitudes exceeding 3000 m, with 41.2% living at ultra-high altitudes (≥ 3500 m). Intergroup analysis indicated that participants from ultra-high altitudes were more susceptible to depression (OR 3.48, 95% CI 1.35–2.91), suggesting that the prevalence of depression may increase with higher altitudes. People who live in high-altitude places encounter unique circumstances typified by low quantities of atmospheric oxygen, which may render them more vulnerable to hypoxia. Inadequate oxygen to the brain can cause cognitive sluggishness and difficulty keeping focus^15^. As well, fast heart rates and rapid breathing caused by low oxygen levels can originate or worsen emotional disorders such as anxiety and sadness. Lower partial pressure of oxygen enhances the excitability of the central nervous system, resulting in poor sleep quality, while chronic insomnia may exacerbate depressive symptoms. According to the monoamine hypothesis of depression^16^ a deficiency of neurotransmitters in the brain, such as dopamine, serotonin, and catecholamines, is a known contributor to depression. Serotonin, also known as an emotional neurotransmitter, is assumed to be associated with well-being and plays an important role in controlling sleep patterns and intestinal function^17^. Hypoxia can inhibit the generation of serotonin in the brain, and low serotonin levels have been linked to depressed symptoms^18^. Furthermore, the difficult natural conditions present in high-altitude areas—characterized by isolation, high levels of UV(ultraviolet) radiation, and cold, dry weather—can readily cause unpleasant emotional states. Harsh natural conditions can significantly impact hippocampal neurogenesis, decrease serotonin bioavailability, disrupt circadian rhythms, and lower melatonin secretion^19^. Notably, melatonin has been associated with depression in both animal models and human studies^20^. The combination of physiological and psychological stressors significantly impacts the mental health of individuals living at high altitudes^21^.

Based on this study, the prevalence of depression increases with age, peaking in the 55–64 age range before gradually descending after the age of 60, regardless of gender. As a result, this particular group is considered high-risk for depression. People in this age group may experience increased anxiety and depression due to the different demands that they faced at work, with their financial situation, and with their families. As people get older, their physiological processes gradually diminish, which can lead to disruptions in the endocrine and neurological systems, severely affecting emotional stability. Moreover, various health conditions common in middle-aged and elderly people, such as hypertension, coronary artery disease, and diabetes, can be linked to depression. Furthermore, prolonged use of some drugs that affect neurotransmitter balance may cause or aggravate depressive symptoms^22^. The rapid pace of social evolution and cultural shifts may have an impact on traditional Tibetan culture, leading to cultural identity crises and psychological stress among older adults. The influence of Han culture on Tibetan Buddhism has caused it to gradually lose its prominence. To protect their faith, middle-aged and older Tibetans have responded by narrowing their social contacts. The emergence of the Internet has also led to a ‘digital divide’ between younger people and their middle-aged and older counterparts, which causes the latter to feel depressed, alone, and powerless^23^. A steady fall in social engagements, as well as a reduction in the number of friends and relatives, may contribute to feelings of loneliness and despair, exacerbating depression symptoms^24^.

Our findings indicate that those with a BMI < 18.5 are more prone to develop depression. Significant evidence indicating a positive correlation between higher BMI levels in individuals of European descent and an increased likelihood of experiencing depression^2^. This relationship can be attributed to both psychological and physiological factors. Obesity can lead to a range of psychological issues and disruptions in gut microbiota due to poor lifestyle habits, such as excessive consumption of sugar and fat. This can increase inflammation, disrupt neurotransmitter activity, and potentially cause depression^25^. Individuals with depression frequently experience more obvious gastrointestinal symptoms than those without depression. Furthermore, the intensity of depression scores is substantially associated with gut-related symptoms^26^.

However, the relationship between obesity and depression can vary by demographic group. Jessica et al.^27^ conducted multiple Mendelian randomization (MR) analyses to analyze the relationship between BMI, waist-to-hip ratio (WHR), and depression in the East Asian population. Their findings indicated an inverse relationship, suggesting that a higher BMI was associated with a lower likelihood of depression. The contrary association discovered in this study contradicts prior findings in European populations. Ancestry and geographic location may help explain these differences. It has been established that those who are underweight have a greater possibility of experiencing depression than those who are of normal weight^28^. This indicates that the relationship between body mass index (BMI) and depression is not merely linear but rather exhibits a ‘U’-shaped curve. Other studies have also corroborated this curvilinear relationship^29^. In addition, there may also be a reciprocal relationship between low BMI and depression. Depression can cause a decrease in appetite and subsequent weight loss, whereas low body weight might increase the risk of depression through a variety of pathways. For example, imbalances in endocrine function, such as those affecting the pituitary and thyroid glands due to malnutrition^30^, alongside deficiencies in vitamin D and omega-3 fatty acids^31^, as well as inflammatory responses caused by impaired immune function, may all contribute to an increased risk of depression.

According to this research, females exhibited a higher prevalence of depression (53.45%) compared to males (42.03%). However, this gender difference was not statistically significant (*P* > 0.05). Some studies have shown that women are more likely than men to develop depression, which could be linked to hormonal differences and women’s heightened sensitivity to various influencing factors^32^. Depression differs by gender, starting from age 11 to 15 and continuing into the reproductive years^33^. Moreover, life stages unique to women—such as pregnancy, the postpartum period, and perimenopause—are associated with an elevated risk of depression. This observation aligns with the prevalence trend identified in this study.

## limitation

This study has several limitations. First, we selected a specific area in the southern Tibetan Plateau and employed convenience sampling rather than random sampling, which could result in sample bias and influence the study’s representativeness. Secondly, the study’s cross-sectional design limits our ability to draw causal inferences. Our subsequent analysis revealed that there are variations in the prevalence of depression when considering different age groups, altitudes, and body mass index (BMI) categories. Despite these observed differences, it remains inconclusive whether age, altitude, and BMI can be definitively identified as direct risk factors for depression. Third, our team utilized questionnaires to assess levels of depression, which may be deemed subjective. Finally, the Qinghai-Tibet Plateau’s distinct climate and dietary practices increase the prevalence of people with high body mass indexes (BMIs) and decrease the sample size of people with low BMIs, potentially resulting in sample bias.

## Conclusion

In conclusion, depression is notably prevalent among people who live at high elevations. Our findings indicate that among Tibetan Plateau residents, depression is significantly associated with higher altitude, lower BMI, and older age. This study provides valuable insights into the psychological health of people living in the southeastern Tibetan Plateau. Future research focusing on the health of local inhabitants should consider the detrimental effects of both the geographical environment and socio-cultural factors on cognitive processes and mental well-being.

## Data Availability

The data that support the findings of this study are available from the corresponding author upon special request.

## Abbreviations

BMI: Body mass index
AOR: Adjusted odds ratios
Cis: Confidence intervals
COVID-19: Coronavirus disease 2019
UV: Ultraviolet
